# MEK Inhibitor U0126 Reverses Protection of Axons from Wallerian Degeneration Independently of MEK–ERK Signaling

**DOI:** 10.1371/journal.pone.0076505

**Published:** 2013-10-04

**Authors:** Catherine Evans, Simon J. Cook, Michael P. Coleman, Jonathan Gilley

**Affiliations:** Signalling Programme, The Babraham Institute, Cambridge, Cambridgeshire, United Kingdom; Massachusetts General Hospital/Harvard Medical School, United States of America

## Abstract

Wallerian degeneration is delayed when sufficient levels of proteins with NMNAT activity are maintained within axons after injury. This has been proposed to form the basis of 'slow Wallerian degeneration' (*Wld* ^S^), a neuroprotective phenotype conferred by an aberrant fusion protein, Wld^S^. Proteasome inhibition also delays Wallerian degeneration, although much less robustly, with stabilization of NMNAT2 likely to play a key role in this mechanism. The pan-MEK inhibitor U0126 has previously been shown to reverse the axon-protective effects of proteasome inhibition, suggesting that MEK-ERK signaling plays a role in delayed Wallerian degeneration, in addition to its established role in promoting neuronal survival. Here we show that whilst U0126 can also reverse Wld^S^-mediated axon protection, more specific inhibitors of MEK1/2 and MEK5, PD184352 and BIX02189, have no significant effect on the delay to Wallerian degeneration in either situation, whether used alone or in combination. This suggests that an off-target effect of U0126 is responsible for reversion of the axon protective effects of Wld^S^ expression or proteasome inhibition, rather than inhibition of MEK1/2-ERK1/2 or MEK5-ERK5 signaling. Importantly, this off-target effect does not appear to result in alterations in the stabilities of either Wld^S^ or NMNAT2.

## Introduction

Effective therapeutic targeting of Wallerian degeneration and other types of axon degeneration that share a common molecular basis (Wallerian-like axon degeneration) could have profound implications for numerous neurodegenerative diseases where axonopathy contributes to pathogenesis [1]. An aberrant fusion protein, Wld^S^, naturally only found in a single mutant mouse (*Wld* ^S^), can delay Wallerian degeneration markedly [2], [3]. Studies of Wld^S^ function have provided considerable insight into the intrinsic mechanisms involved in the process and have recently led to the identification of a number of key regulatory molecules and pathways [4]–[14]. This includes the finding that NMNAT2, which shares critical nicotinamide mononucleotide adenylyltransferase (NMNAT) activity with Wld^S^, is an endogenous axon maintenance factor, with depletion of NMNAT2 in axons likely acting as a trigger for degeneration [6]. Despite being predominantly nuclear, a small pool of axonal Wld^S^ appears to be responsible for protection [15]–[17]. Consequently, because Wld^S^ is much more stable than very short-lived NMNAT2, it has been suggested that it delays axon degeneration by directly substituting for NMNAT2 loss in compromised axons [6]. However, the relationship between NMNAT activity and other regulators and/or executers of the degeneration pathway has yet to be fully established.

Canonical MEK1/2-ERK1/2 signaling, and more recently MEK5-ERK5 signaling, have been shown to be critical for neuronal stress responses and/or neurotrophin-mediated neuronal survival [18]–[20]. However, a study using the pan-MEK inhibitor U0126 has also implicated MEK-ERK signaling in the protection against injury-induced or developmental axon degeneration after proteasome inhibition [21]. NMNAT2 levels are stabilized after proteasome inhibition [6], providing one possible explanation for delayed axon degeneration under these conditions. We therefore assessed the effects of U0126 on the *Wld* ^S^ phenotype to test the hypothesis that NMNAT activity keeps axons healthy by sustaining MEK-ERK signaling. As U0126 can inhibit both MEK1/2 and MEK5 [22], [23], we also used more specific small molecule inhibitors of MEK1/2 and MEK5 to differentiate the roles of these pathways in relation to axon protection. Surprisingly, our results appear to rule out involvement of either target.

## Results

### U0126 can revert the slow Wallerian degeneration (*Wld* ^S^) phenotype

The ability of the pan-MEK inhibitor U0126 (at 50 µM) to reverse delayed Wallerian degeneration of neurites in rat superior cervical ganglion (SCG) cultures after proteasome inhibition suggested that MEK-ERK signaling might play an important role in axon maintenance [21]. We therefore investigated whether MEK-ERK signaling also contributes to the delay of Wallerian degeneration in *Wld* ^S^ neurons.

**Figure 1:**
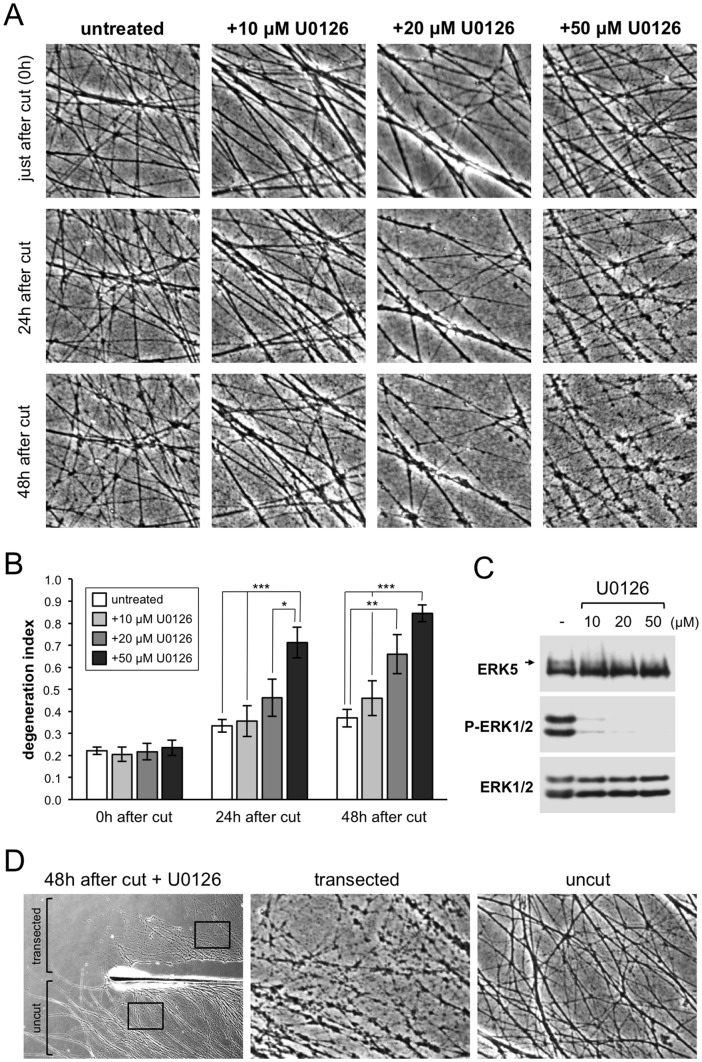
Reversion of the *Wld* ^S^ phenotype by MEK inhibitor U0126. (A) Representative phase contrast images of transected neurites in *Wld* ^S^ SCG explant cultures treated with different concentrations of U0126 as indicated (untreated  =  DMSO). Images of the same field of distal neurites were captured at the times after transection shown on the left. (B) Quantification of neurite degeneration for transected *Wld* ^S^ neurites as in (A). Degeneration index (± SEM) was calculated from multiple fields in n = 4 independent experiments. * *p*<0.05, ** *p*<0.01, and *** *p*<0.001, 2-way repeated measures ANOVA with Tukey's multiple comparisons post hoc tests. All statistically significant differences are marked. All other comparisons were not significant. (C) Representative immunoblots showing inhibition of ERK1/2 and ERK5 phosphorylation in whole *Wld* ^S^ SCG explant cultures (cell bodies and neurites combined) 48 hours after treatment with different concentrations of U0126. Phosphorylated ERK1/2 was detected using a phosphorylation-dependent antibody. Phosphorylated ERK5 was detected as a slower migrating band (indicated by an arrow) using a phosphorylation-independent antibody. Total ERK1/2 and ERK5 levels act as sample references. (D) Representative phase contrast image (left) showing cut and uncut neurites in *Wld* ^S^ SCG explant cultures 48 hours after transection and treatment with 50 µM U0126. Boxed regions are magnified to show morphology of cut and uncut neurites.

Neurites in SCG explant cultures from *Wld* ^S^ mice are protected from Wallerian degeneration for at least 72 hours after being separated from their cell bodies. In contrast, transected wild-type neurites begin to degenerate after a short latent phase of just 4–6 hours [6], [12]. We found that 50 µM U0126 partially reverted the slow Wallerian degeneration phenotype of cut *Wld* ^S^ neurites (Figure 1A and 1B). Neurites treated with U0126 consistently showed physical signs of degeneration by 24 hours after cut, whereas untreated transected neurites remained healthy for at least 48 hours, as expected. Intriguingly, we found that the ability of U0126 to revert the *Wld* ^S^ phenotype appeared highly dose-dependent but did not fully correlate with inhibition of MEK1/2-ERK1/2 signaling. ERK1/2 phosphorylation was robustly inhibited by both 10 µM and 20 µM U0126 (Figure 1C) even though these doses were much less effective at reverting Wld^S^-mediated neurite protection (Figure 1A and 1B). Uncut *Wld* ^S^ neurites treated with U0126 remained healthy over the same time-course (Figure 1D) indicating that this effect was specific to severed neurites.

Importantly, U0126 can also inhibit the MEK5-ERK5 signaling pathway [22], [23], which is functional in this type of neuron [18]. Consistent with this, we noted a reduction in the proportion of ERK5 showing retarded electrophoretic mobility after U0126 treatment in these experiments (Figure 1C). Efforts were made to assess changes in ERK5 phosphorylation directly, but none of the phosphorylation-dependent antibodies tested were sensitive enough to specifically detect endogenous levels of the phosphorylated protein. However, retarded electrophoretic mobility of ERK5 has previously been used as an indicator of ERK5 phosphorylation in SCG neurons [18], and phosphorylation of the TEY motif in the ERK5 activation loop correlates with retarded ERK5 electrophoretic mobility in extracts from cell lines overexpressing components of the MEK5-ERK5 pathway [24]. Interestingly, we found that in addition to full-length ∼110 kDa ERK5 (Figure 1C), SCG neurons also express a ∼60 kDa truncated form of the protein, ERK5-T, which is the result of alternative splicing [25] (Figure S1). Like full-length ERK5, this truncated variant can be phosphorylated by MEK5 [25], but we have so far been unable to detect this in SCG neurons, and consequently determine its sensitivity to U0126 (data not shown). Nevertheless, the fact that U0126 inhibits phosphorylation of full-length ERK5 at concentrations that effectively revert Wld^S^-mediated axon protection meant that MEK5-ERK5 signaling, either alone or in combination with MEK1/2-ERK1/2 signaling, could mediate the effects of Wld^S^.

### Combined inhibition of MEK1/2 and MEK5 with PD184352 and BIX02189 does not replicate the effects of U0126

**Figure 2:**
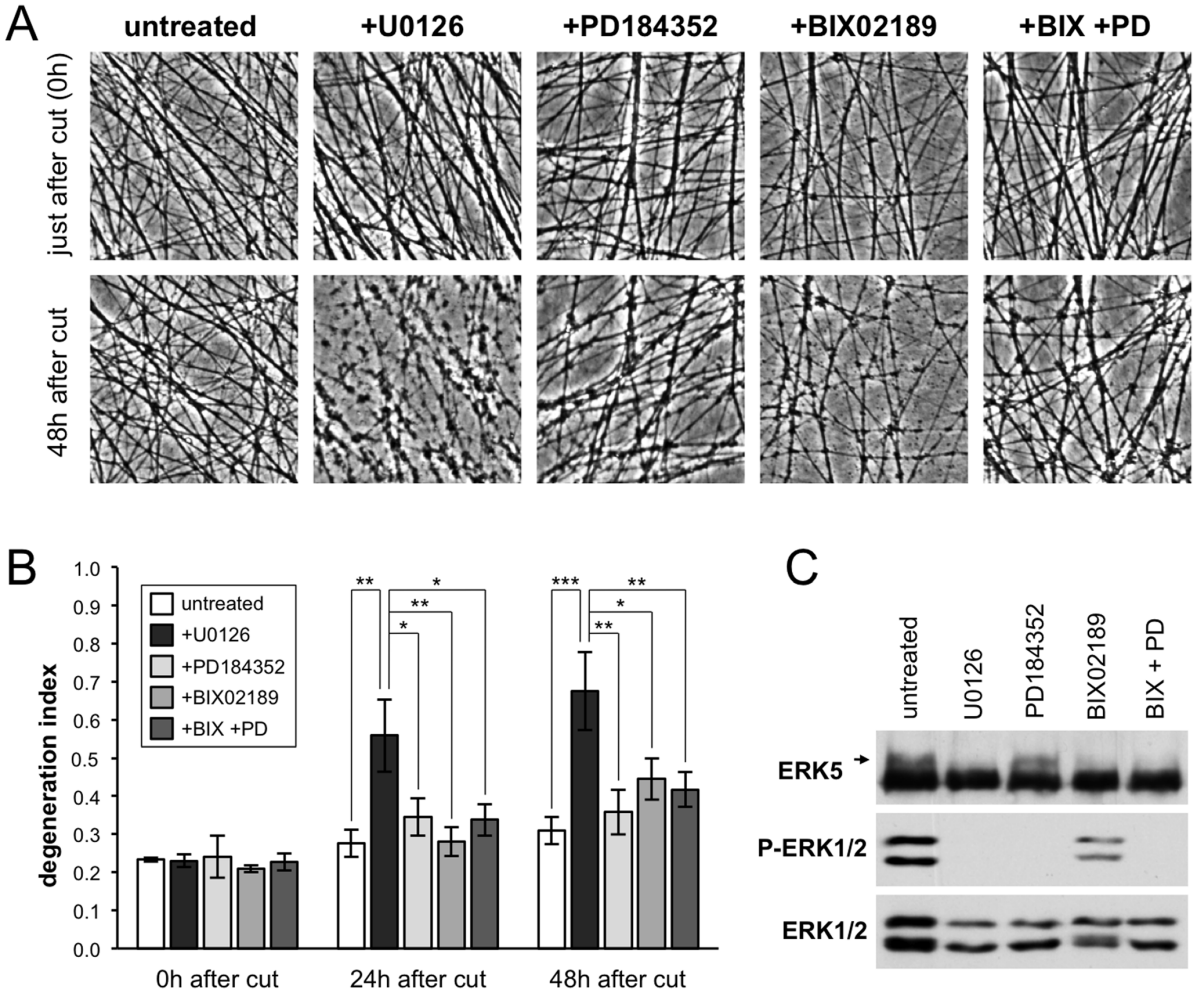
MEK1/2 inhibitor PD184352 and MEK5 inhibitor BIX02189 fail to revert the *Wld* ^S^ phenotype. (A) Representative phase contrast images of transected neurites in *Wld* ^S^ SCG explant cultures treated with U0126 (50 µM), PD184352 (5 µM) and/or BIX02189 (10 µM) as indicated (untreated  =  DMSO). Images of the same field of distal neurites were captured just after transection and 48 hours later. (B) Quantification of neurite degeneration for transected *Wld* ^S^ neurites as in (A). Degeneration index (± SEM) was calculated from multiple fields in n = 3 or 4 independent experiments. * *p*<0.05, ** *p*<0.01 and *** *p*<0.001, 2-way repeated measures ANOVA with Tukey's multiple comparisons post hoc tests. All statistically significant differences are marked. All other comparisons were not significant. (C) Representative immunoblots showing inhibition of ERK1/2 and ERK5 phosphorylation in whole *Wld* ^S^ SCG explant cultures (cell bodies and neurites combined) after 48 hours treatment with U0126 (50 µM), PD184352 (5 µM) and/or BIX02189 (10 µM) as indicated (untreated  =  DMSO). Phosphorylated ERK1/2 was detected using a phosphorylation-dependent antibody. Phosphorylated ERK5 was detected as a slower migrating band (indicated by an arrow) using a phosphorylation-independent antibody. Total ERK1/2 and ERK5 levels act as sample references.

In order to define the relative contributions of MEK1/2-ERK1/2 and MEK5-ERK5 signaling to the slow Wallerian degeneration (*Wld* ^S^) phenotype we used other, more selective MEK inhibitors as alternatives to U0126. PD184352 is a highly selective inhibitor of MEK1/2 [23], [26], whilst BIX02189 has recently been identified as a selective inhibitor of MEK5/ERK5 signaling that fails to inhibit MEK1/2 [27]. Surprisingly, we found that, unlike U0126, PD184352 and BIX02189 whether alone, or in combination, did not significantly accelerate degeneration of transected *Wld* ^S^ neurites (Figure 2A and 2B). This was despite the fact that we confirmed (a) maximal inhibition of each pathway at the concentrations of PD184352 and BIX02189 used and (b) each drug exhibited the expected selectivity (Figure 2C). These results indicated that U0126 reverses the slow Wallerian degeneration (*Wld* ^S^) phenotype through a mechanism that must be largely independent of MEK1/2-ERK1/2 or MEK5-ERK5 signaling.

**Figure 3:**
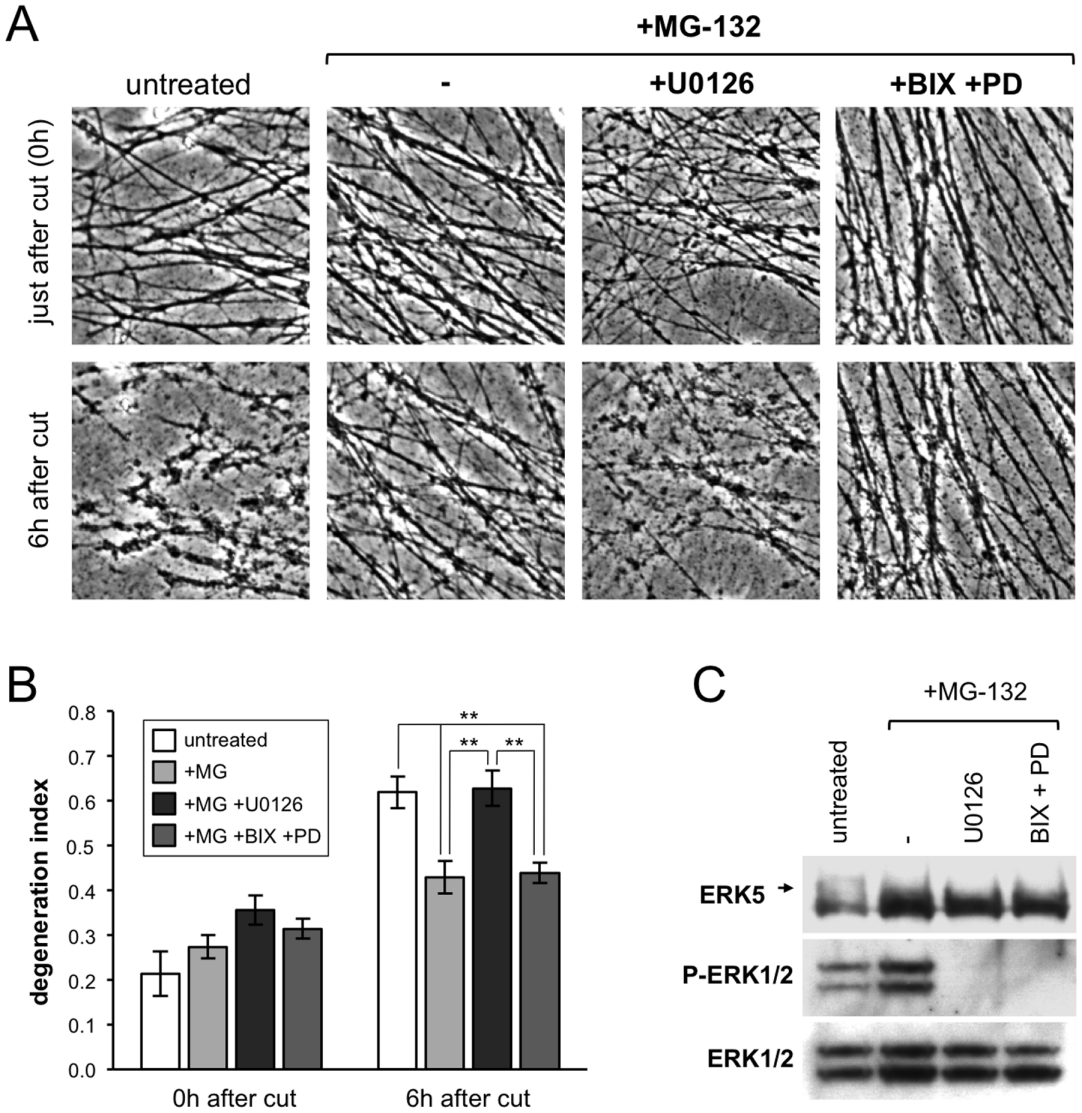
Delayed Wallerian degeneration after proteasome inhibition is not reverted by PD184352 and BIX02189 combined. (A) Representative phase contrast images of transected wild-type neurites in SCG explant cultures treated with U0126 (50 µM), or PD184352 (5 µM) plus BIX02189 (10 µM) combined, after proteasome inhibition by MG-132 (20 µM) (untreated  =  DMSO). Images of the same field of distal neurites were captured just after transection and 6 hours later. (B) Quantification of neurite degeneration for transected neurites as in (A). Degeneration index (± SEM) was calculated from multiple fields in n = 3 independent experiments. ** *p*<0.01, 2-way repeated measures ANOVA with Tukey's multiple comparisons post hoc tests. All statistically significant differences are marked. All other comparisons were not significant. (C) Representative immunoblots showing inhibition of ERK1/2 and ERK5 phosphorylation in whole wild-type SCG explant cultures (cell bodies and neurites combined) 9 hours after proteasome inhibition by MG-132 (20 µM) and 6 hours after treatment with U0126 (50 µM), or a combination of PD184352 (5 µM) plus BIX02189 (10 µM) (untreated  =  DMSO). Phosphorylated ERK1/2 was detected using a phosphorylation-dependent antibody. Phosphorylated ERK5 was detected as a slower migrating band (indicated by an arrow) using a phosphorylation-independent antibody. Total ERK1/2 and ERK5 levels act as sample references.

In light of this result, we confirmed the U0126-mediated reversion of delayed Wallerian degeneration after proteasome inhibition reported previously [21] and assessed whether this was also independent of MEK-ERK signaling. Inhibition of proteasome function with MG-132 three hours prior to cutting resulted in protection of transected wild-type neurites for at least 6 hours (Figure 3A and 3B), consistent with previous reports [6], [12]. Although the delay in degeneration in this system appears less robust than that reported using other read-outs [21], we nevertheless found that U0126 similarly reversed the protection (Figure 3A and 3B). In contrast, the combined action of PD184352 and BIX02189 had no significant effect (Figure 3A and 3B), despite again inhibiting ERK1/2 and ERK5 phosphorylation robustly (Figure 3C). Therefore, our findings suggest that the ability of U0126 to reverse delayed neurite degeneration after proteasome inhibition also occurs via a mechanism that is independent of MEK-ERK signaling.

### U0126 does not alter the stability of NMNAT2 or Wld^S^

**Figure 4:**
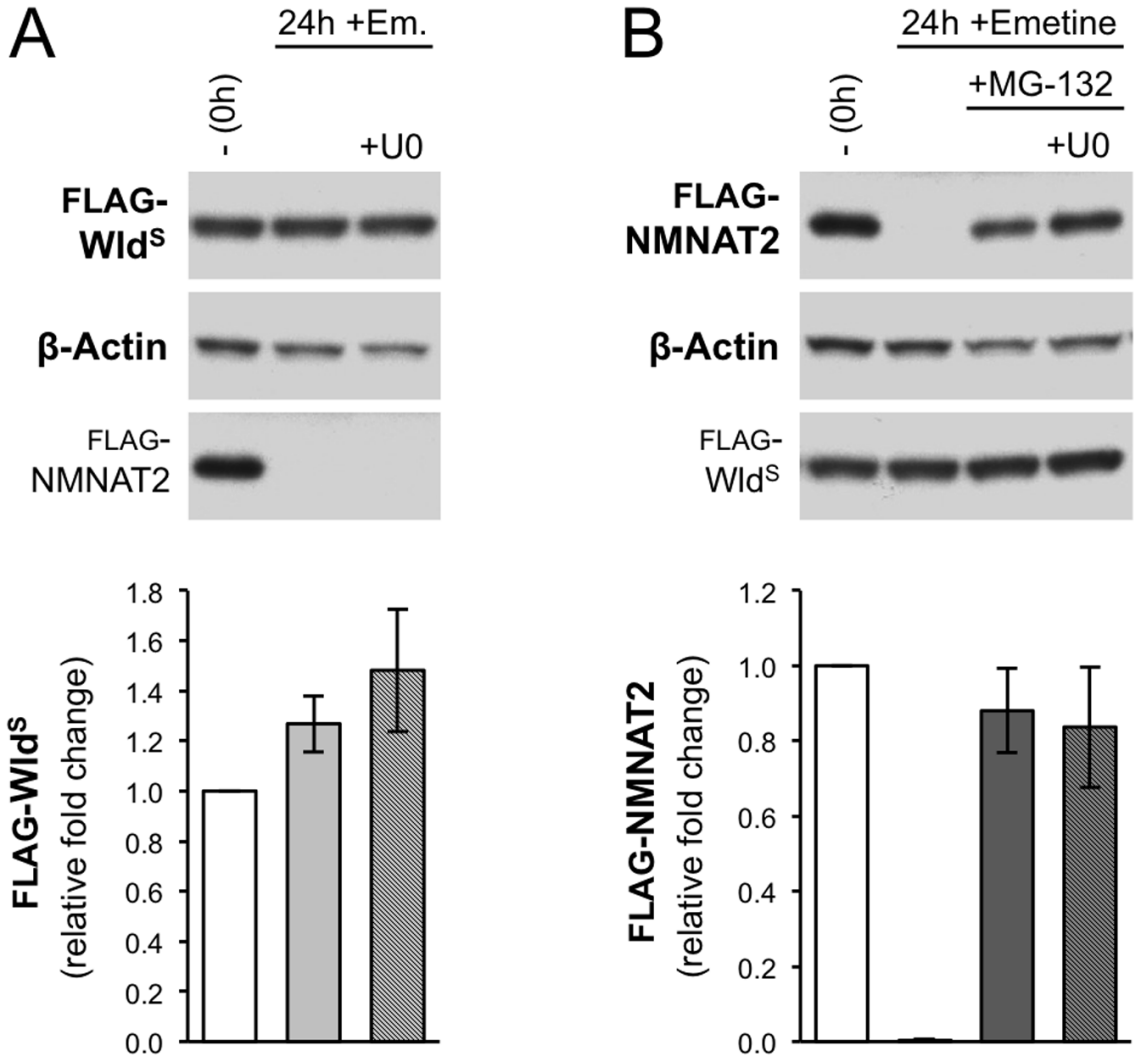
U0126 does not alter FLAG-NMNAT2 or FLAG-Wld^S^ turnover in transfected HEK 293T cells. Immunoblot analyses assessing the effects of U0126 (50 µM) on natural turnover of FLAG-Wld^S^ (A) and stabilization of FLAG-NMNAT2 after proteasome inhibition (B) in HEK 293T cells. Cells were co-transfected with FLAG-Wld^S^ and FLAG-NMNAT2 expression constructs. 24 hours after transfection cells were treated with emetine (10 µM), together with proteasome inhibitor MG-132 (20 µM) and/or U0126 (50 µM), as indicated, for a further 24 hours. Control cells (–) treated with DMSO were collected at the time of emetine addition (0 hours) to act as a reference for expression levels before protein synthesis was blocked. ß-Actin acts as the sample reference. Blots of FLAG-NMNAT2 and FLAG-Wld^S^ in (A) and (B) respectively (bottom panels) are included only as controls (for transfection efficiency or emetine efficacy). Representative images are shown. Relative mean levels (± SEM) of FLAG-Wld^S^ (A) and FLAG-NMNAT2 (B) are shown below the relevant lanes on each blot after normalization to ß-Actin (based on data from n = 2 and n = 4 respectively). Data are presented relative to the DMSO controls (–) (set at 1).

Reduced turnover of short-lived NMNAT2 has been correlated with the delay of Wallerian degeneration after proteasome inhibition in SCG explant cultures and we proposed that Wld^S^ protects neurites because it is relatively much more stable [6]. We therefore investigated whether U0126 accelerates turnover of Wld^S^ or NMNAT2 to account for its effects on neurite preservation. We assessed protein stability in a HEK 293T cell culture-based transfection assay employing a protein synthesis block which broadly reflects rates of turnover in SCG neurites [6]. We found no evidence for increased turnover of FLAG-Wld^S^ following U0126 treatment (Figure 4A). In addition, proteasome inhibition stabilized very short-lived FLAG-NMNAT2 after protein synthesis inhibition, as expected, but this stabilization was not altered by U0126 (Figure 4B). Therefore, it appears unlikely that U0126 reverses the slow Wallerian degeneration phenotype, or delayed Wallerian degeneration after proteasome inhibition, by reducing levels of Wld^S^ or NMNAT2 respectively.

## Discussion

A protective or maintenance role for MEK-ERK signaling in injured axons and during developmental axon pruning was previously postulated based on the negative effects of the pan-MEK inhibitor U0126 on neurite health when modeling these conditions in primary rat SCG cultures [21]. Our finding that U0126 similarly reverses Wld^S^-mediated protection of severed mouse SCG neurites initially appeared to support this general conclusion. However, use of more selective MEK1/2 and MEK5 inhibitors demonstrated that the effects of U0126, both in injured *Wld* ^S^ neurites and injured wild-type neurites after proteasome inhibition, are independent of MEK/ERK signaling and must therefore be a consequence of an off-target effect.

Many small molecule kinase inhibitors have unexpected off-target effects on unrelated kinases [28]-[30]. Whilst U0126 appears relatively selective towards MEKs [29], [30], the panels of kinases tested, whilst extensive, were not complete. Therefore the effects of U0126 seen in this study could be due to as yet uncharacterized off-target kinase inhibition. We have already provisionally ruled out reductions in steady-state levels of Wld^S^ or levels of NMNAT2 after proteasome inhibition as downstream consequences of such off-target inhibition, given their critical axonal survival and maintenance functions. Interestingly, U0126 has previously been shown to reduce ATP levels in cultured cells resulting in an increased AMP:ATP ratio and activation of AMPK via what appears to be a MEK-independent mechanism [31]–[33]. Since declining ATP levels might contribute to the initiation or execution of Wallerian degeneration [34], a U0126-mediated reduction in ATP could thus account for its effects on preservation of transected axons, although PD184352 may have similar off-target effects in some cell types [31]. Interestingly, mitochondrial ATP production and Ca^2+^ buffering have respectively been shown to be enhanced in *Wld* ^S^ mice and in transgenic flies expressing Wld^S^ [35], [36]. Given the established link between mitochondrial Ca^2+^ levels and ATP generation [37], this raises the possibility that the critical off-target effect of U0126 in this study might be to influence mitochondrial Ca^2+^ homeostasis in some way. However, a recent report suggesting that mitochondria are not required for Wld^S^-mediated axon protection in flies [38], seems to challenge this idea.

Our finding that U0126 does not affect the short-term maintenance and survival of uninjured *Wld* ^S^ neurites is in agreement with the previous finding that it only impacts delayed degeneration of severed or otherwise compromised wild-type neurites [21]. Irrespective of any off-target effects of U0126, this clearly indicates that loss of ERK1/2 and ERK5 signaling is not sufficient to induce spontaneous degeneration of intact neurites. Declining ERK1/2 phosphorylation, which appears to precede loss of total ERK1 in transected wild-type neurites [21], could nevertheless still contribute to the progression of Wallerian degeneration, but the possibility that this is simply an early consequence of the degeneration process itself also cannot be ruled out.

We conclude that MEK-ERK signaling, specifically through MEK1/2 or MEK5, is not required for the preservation of transected neurites by Wld^S^ or proteasome inhibition. Rather, the widely-used MEK inhibitor U0126 appears to reverse this protection via an as yet unidentified target. Importantly, this study highlights the risk of interpreting results based solely on data obtained with this compound. Reassessment of findings using more selective MEK1/2 and MEK5 inhibitors, such as PD184352 and BIX02189, should be performed as standard and could lead to important new insights into cellular signaling.

## Materials and Methods

### Ethical treatment of animals

All animal work was carried out in strict accordance with the UK Animals (Scientific Procedures) Act, 1986, under Project Licenses PPL 80/1778 and 80/2254 and was approved by the Babraham Institute Animal Welfare, Experimentation and Ethics Committee. Postnatal day 1 or 2 (P1 or P2) mouse pups were sacrificed by decapitation, with every effort made to limit suffering.

### Cell culture

Superior cervical ganglia were dissected from P1 or P2 mouse pups. Cleaned explants were placed in the center of 3.5 cm tissue culture dishes pre-coated with poly-L-lysine (20 µg/ml for 1–2 hours; Sigma) and laminin (20 µg/ml for 1–2 hours; Sigma). Explants were cultured in Dulbecco's Modified Eagle's Medium (DMEM) with 4500 mg/L glucose and 110 mg/L sodium pyruvate (Sigma), 2 mM glutamine, 1% penicillin/streptomycin, 100 ng/ml 7S NGF (all Invitrogen), and 10% fetal bovine serum (Sigma). 4 µM aphidicolin (Calbiochem) was used to reduce proliferation and viability of non-neuronal cells. Experiments were performed after 5–7 days *in vitro*.

C57BL/6JOlaHsd and homozygous C57BL/6OlaHsd-Wld (*Wld* ^S^) mice were originally obtained from Harlan UK (Bicester, UK) and maintained as a long-term breeding colony at the Babraham Institute.

HEK 293T cells were cultured under standard conditions in DMEM with 4500 mg/L glucose and 110 mg/L sodium pyruvate (PAA), supplemented with 2 mM glutamine and 1% penicillin/streptomycin (both Invitrogen), and 10% fetal bovine serum (Sigma).

### Inhibitor treatments

The MEK inhibitors U0126 (Promega), PD184352 (Selleck), and BIX02189 (a kind gift from Roger Snow, Boehringer Ingelheim) were dissolved in DMSO as 10 mM stock solutions and diluted in media as required. InSolution MG-132 (Calbiochem) was added to SCG explant cultures at a final concentration of 20 µM 3 hours prior to neurite transection. This pre-treatment is required to see neurite protection in these cultures [6], [12].

### Quantification of transected neurite degeneration

Neurites were cut with a disposable scalpel roughly half-way between their cell bodies and their most distal ends. Inhibitors or vehicle (DMSO) were added to the media less than 10 minutes before transection. Phase contrast images of transected neurites were captured on an Olympus IX81 inverted microscope using a Soft Imaging Systems (SIS) F-View camera linked to a PC running the appropriate SIS imaging software and 10x or 20x objectives. Images of the same field of transected neurites were captured at different time points after cut. Images were processed for manuscript presentation using Adobe Photoshop Elements 4.0. Neurite degeneration was quantified as a Degeneration Index essentially as described previously [39], except that for calculating the area representing degenerated axon fragments using the ImageJ Particle Analyzer function a size range of 20–350 pixels was used for images captured with the 20x objective (total image size  =  1376×1032 pixels).

### Immunoblot analysis

Whole SCG explant extracts were collected at the end of each experiment as indicated in the figure legends. Cell bodies and proximal neurites were combined with transected neurites to provide sufficient non-degenerated material for assessment of the effects of inhibitors on ERK1/2 and ERK5 phosphorylation. HEK 293T cells at 60–80% confluence in 12-well plates were co-transfected with 100 ng FLAG-Wld^S^ and 250 ng FLAG-NMNAT2 expression constructs (described previously [6]) per well using Lipofectamine 2000 reagent (Invitrogen). Twenty-four hours after transfection cells were treated with inhibitors as described. In both cases cells were washed in cold PBS containing cOmplete Mini protease inhibitor cocktail (Roche) prior to lysis directly into 2x Laemmli sample buffer.

Extracts were separated by standard SDS-PAGE on 6 or 12% gels (depending on the proteins being detected) and transferred to Immobilon-P membrane (Millipore) or nitrocellulose (for ERK5 detection) using the Bio-Rad Mini-PROTEAN III wet transfer system. Blots were blocked and incubated with primary antibodies overnight at 4°C in 1x TBS p.H. 8.3 with 0.05% Tween 20 and 5% milk powder, followed by the appropriate HRP-conjugated secondary antibody (1 hour room temperature at 1∶2000–1∶5000) and detection by ECL or ECL plus (GE Healthcare), with washes between each stage. Antibody-specific instructions provided by the supplier were followed for detection of ERK5. The following primary antibodies were used; mouse monoclonal anti-FLAG M2 (1:2000, Sigma), rabbit polyclonal Wld18 anti-Wld^S^ (1:4000), mouse monoclonal anti-ß-Actin clone AC-74 (1∶5000, Sigma A5316), rabbit polyclonal anti-BMK1/ERK5 (1∶750, Upstate/Millipore 07-039), mouse monoclonal anti-ERK1 (1∶2000, BD Transduction Laboratories 610031) which also recognizes ERK2, and mouse monoclonal anti-phospho-ERK1/2 (1∶2000, Cell Signaling Technology 9106).

### Statistical analysis

Data are presented as mean ± SEM. The statistical analyses described in the text were performed using Microsoft Excel and Prism (GraphPad Software Inc., La Jolla, CA, USA) software. Differences were considered statistically significant if *p*<0.05.

## Supporting Information

Figure S1 **SCG neurons express the truncated ERK5 splice variant, ERK5-T.** (A) *Erk5* mRNA encodes 806 amino acid ERK5. Failure to remove intron 4 in the *Erk5-T* splice variant introduces an alternative termination codon resulting in a truncated protein of 502 amino acids that shares the N-terminal 492 amino acids with ERK5 (protein lengths indicated here do not include the termination codon) [25]. (B) RT-PCR analysis of SCG mRNA using primers flanking intron 4 in *Erk5-T* mRNA (5'-CCTCCAGCACTGCCACCAT-3' and 5'-CGCTTCTCTTCTCGTTCTCG-3'). A product of 260 bp was amplified from *Erk5* mRNA, lacking the 103 bp intron 4, and a product of 363 bp was amplified from *Erk5-T* mRNA. *Erk5-T* mRNA appears to be significantly less abundant than *Erk5* mRNA. RT-PCR was performed as described previously [6]. (C) Immunoblot analysis using antibodies (Biosource MBS615166 and SantaCruz ERK5 N-19) raised against conserved epitopes in ERK5-T (shared with ERK5). A ∼60 kDa band, corresponding to the expected size of ERK5-T, was detected by both antibodies in the SCG extract but was absent from *Erk5^−/−^* mouse embryo fibroblasts (MEFs). Both antibodies failed to detect endogenous levels of full-length ERK5 in the SCG extract (even though *Erk5* mRNA appears more abundant than *Erk5-T* mRNA), but did detect stably overexpressed exogenous ERK-5 (HA-tagged). Both antibodies cross-reacted with several non-specific bands. The most intense cross-reacting bands are marked (*). A different antibody (Upstate/Millipore, 07-039) was used to detect full-length ERK5 in SCGs (Figures 1, 2 and 3). This was raised against C-terminal amino acids (783-806) in human ERK5 that are not present in ERK5-T.
(TIF)
