# Supplementary figures and images for: MEK Inhibitor U0126 Reverses Protection of Axons from Wallerian Degeneration Independently of MEK–ERK Signaling
Source: PLoS One. 2013 Oct 4;8(10):e76505. doi: 10.1371/journal.pone.0076505 (PMC3790678; doi:10.1371/journal.pone.0076505)

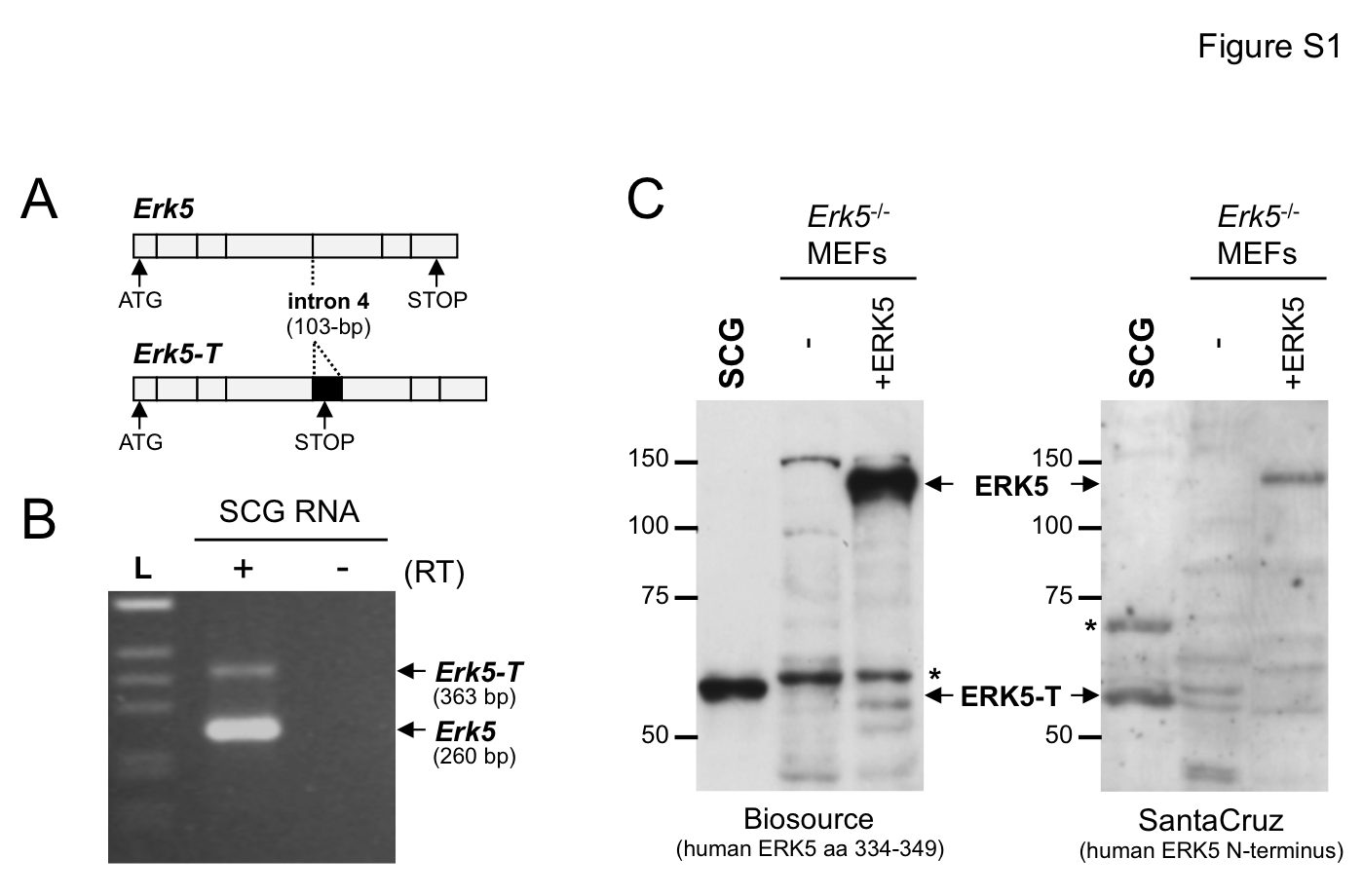

Supplement: Figure S1 — SCG neurons express the truncated ERK5 splice variant, ERK5-T. (A) Erk5 mRNA encodes 806 amino acid ERK5. Failure to remove intron 4 in the Erk5-T splice variant introduces an alternative termination codon resulting in a truncated protein of 502 amino acids that shares the N-terminal 492 amino acids with ERK5 (protein lengths indicated here do not include the termination codon) [25]. (B) RT-PCR analysis of SCG mRNA using primers flanking intron 4 in Erk5-T mRNA (5'-CCTCCAGCACTGCCACCAT-3' and 5'-CGCTTCTCTTCTCGTTCTCG-3'). A product of 260 bp was amplified from Erk5 mRNA, lacking the 103 bp intron 4, and a product of 363 bp was amplified from Erk5-T mRNA. Erk5-T mRNA appears to be significantly less abundant than Erk5 mRNA. RT-PCR was performed as described previously [6]. (C) Immunoblot analysis using antibodies (Biosource MBS615166 and SantaCruz ERK5 N-19) raised against conserved epitopes in ERK5-T (shared with ERK5). A ∼60 kDa band, corresponding to the expected size of ERK5-T, was detected by both antibodies in the SCG extract but was absent from Erk5−/− mouse embryo fibroblasts (MEFs). Both antibodies failed to detect endogenous levels of full-length ERK5 in the SCG extract (even though Erk5 mRNA appears more abundant than Erk5-T mRNA), but did detect stably overexpressed exogenous ERK-5 (HA-tagged). Both antibodies cross-reacted with several non-specific bands. The most intense cross-reacting bands are marked (*). A different antibody (Upstate/Millipore, 07-039) was used to detect full-length ERK5 in SCGs (Figures 1, 2 and 3). This was raised against C-terminal amino acids (783-806) in human ERK5 that are not present in ERK5-T. (TIF) [file pone.0076505.s001.tif]
